# Gender Differences in Obesogenic Behaviour, Socioeconomic and Metabolic Factors in a Population-based Sample of Iranians: The IHHP Study

**DOI:** 10.3329/jhpn.v28i6.6609

**Published:** 2010-12

**Authors:** Ali Akbar Tavassoli, Mojgan Gharipour, Alireza Khosravi, Roya Kelishadi, Zahra Dana Siadat, Ahmad Bahonar, Gholam Hosein Sadri, Masoumeh Sadeghi, Katayoun Rabiei, Firouzeh Sajjadi, Sonia Zarfeshani, Babak Eshrati, Shahin Shirani, Nizal Sarrafzadegan

**Affiliations:** ^1^ Cardiology Department, Isfahan University of Medical Sciences, Isfahan, Iran; ^2^ Isfahan Cardiovascular Research Center, Isfahan University of Medical Sciences, Isfahan, Iran; ^3^ Department of Community Medicine, Isfahan University of Medical Sciences, Isfahan, Iran; ^4^ Isfahan Provincial Health Office, Isfahan University of Medical Sciences, Isfahan, Iran; ^5^ Nutrition Department, Isfahan Cardiovascular Research Center, Isfahan University of Medical Sciences, Isfahan, Iran; ^6^ Department of Community Medicine, Arak University of Medical Sciences, Arak, Iran

**Keywords:** Cardiovascular diseases, Cross-sectional studies, Lifestyle, Obesity, Risk factors, Socioeconomic factors, Iran

## Abstract

This study investigated the gender differences in association of some behavioural and socioeconomic factors with obesity indices in a population-based sample of 12,514 Iranian adults. The mean body mass index (BMI), waist circumference (WC), and the waist-to-hip ratio (WHR) were significantly higher in women than in men. Current and passive smoking had an inverse association with BMI among males whereas current smoking, transportation by a private car, and longer duration of watching television (TV) had a positive association with BMI among females. Current and passive smoking, cycling, and Global Dietary Index (GDI) had an inverse association with WC among males. Higher consumption of fruits and vegetables, current and passive smoking, duration of daily sleep, and GDI had an inverse association with WC among females. Using a private car for transportation had a significant positive association with WHR among both males and females. Living in an urban area, being married, and having a higher education level increased the odds ratio of obesity among both the genders. Non-manual work also increased this risk among males whereas watching TV and current smoking increased this risk among females. Such gender differences should be considered for culturally-appropriate interventional strategies to be implemented at the population level for tackling obesity and associated cardiometabolic risk factors.

## INTRODUCTION

Similar to many other developing countries, the Eastern Mediterranean region has experienced a significant rise in cardiovascular diseases (CVDs) in the last two decades (1). According to the estimate of the World Health Organization (WHO), by 2010, CVDs will be the major cause of morbidity and mortality in developing countries (2). Despite the recent improvements in health services which have enhanced the longevity of cardiovascular patients, CVDs are still the leading cause of death in Iran. Changes in lifestyle of the Iranians, including their improper nutritional habits, physical inactivi-ty, and tobacco-use, are said to be responsible for this rise (3).

It is well-documented that obesity is one of the most important and modifiable risk factors of CVDs (4). Several lifestyle behaviours and factors relating to socioeconomic status (SES) may influence this emerging health problem. This influence may differ in terms of gender. To prepare and carry on effective programmes targeting the risk factors for CVDs, reliable data on different characteristics of the population are necessary. The baseline survey of a community-based programme titled Isfahan Healthy Heart Programme (IHHP) assessed the mean levels and prevalence of risk factors for CVDs in three cities in the central part of Iran. Further, it aimed to determine the behavioural and SES factors associated with CVDs to plan necessary population-based interventions.

The present study reports the differences in the obesity indices, other risk factors for CVDs, lifestyle behaviours, and SES among Iranian men and women.

## MATERIALS AND METHODS

### Participants

The methods of the IHHP have been previously described in details (5–6), and here we describe those in brief.

We selected 12,514 men and women, aged ≥19 years, from Isfahan, Najaf-Abad, and Arak—three counties located in the central part of Iran, using multistage random-cluster sampling. Pregnant women and those who were not mentally competent were excluded from the study. Data were collected in different sessions comprising a 30-minute home-interview and physical examinations in clinics. The validated questionnaire included questions on demographic characteristics, smoking, nutrition, and physical activity-related behaviours.

Nutritional behaviours are presented as Global Dietary Index (GDI) that was calculated using a large volume of data obtained on dietary habits through a validated qualitative 48-item food-frequency questionnaire which was adapted from the validated Countrywide Integrated Non-communicable Disease Intervention (CINDI) programme (7). This questionnaire focused on total and saturated fat intake. Frequency responses were scored as 2, 1, or 0 depending on nutritional value, with higher score indicating higher total and saturated fat intakes, e.g. by questions as “How many times a week do you usually eat meat?”

### Definitions

Healthful nutritional behaviour was defined as having more than five times of fruits and vegetables per day (8–9). Physical activity was assessed quantitatively with a detailed questionnaire that assessed the walking or cycling time.

Smoking was defined as current smoking for those who smoke at least one cigarette a day; passive smoking for those who had involuntary inhalation by a non-smoker; and ex-smoker as those who do not actually smoke but have a history of previous regular use of tobacco (10).

Weight and height were measured with calibrated instruments and under a standard protocol (4). Body mass index (BMI) was calculated as weight (kg)/height (m)^2^. Waist circumference (WC) and hip circumference were measured and recorded in cm using standard WHO methods. The waist-to-hip ratio (WHR) was calculated by dividing the circumference of the waist to that of the hip.

Fasting venous blood samples were examined for total cholesterol (TC), high-density lipoprotein (HDL-C), triglycerides (TG), and fasting blood glucose (FBG). Low-density lipoprotein-cholesterol (LDL-C) was calculated by Friedwald equation when TG was less than 400 mg/dL (11). TC was measured using enzymatic colorimetric methods. HDL-C was determined after dextran sulphate-magnesium chloride precipitation of non-HDL-C (12–13). Due to financial limitations, we could not examine the apolipoproteins of all individuals; however, we randomly selected a subsample of 2,000 participants to determine serum apolipoprotein A (apoA), apolipoprotein B (apoB) (Pars Azmon commercial kits), and insulin resistance (Monobind commercial kit by ELISA method).

All the tests were performed in the central laboratory of the Isfahan Cardiovascular Research Center and using auto-analyzer ELAN (Ependorf 2000). For quality-control measures, this laboratory meets the criteria of the national standard laboratory (a WHO collaborating centre in Tehran). Further, it is under external standardization with the central laboratory at the University Hospital Leuven, Belgium.

Marital status, number of family members, having multi-jobs and type of job (private or government), education, income (low, middle, and high), and ownership of car were considered SES dimensions.

Education was assessed by the highest achieved degree. We classified participants into three categories according to the highest achieved degree: primary (8–9 years of primary school), secondary (completed secondary school with final examination), and university (completed degree).

GDI, consumption of vegetables and fruits, type of transportation (public or private), smoking habits, and daily leisure-time and physical activity were assessed as lifestyle behaviours.

### Statistical analysis

Data are expressed as mean±standard deviation (SD). The differences between the mean values were compared by independent *t*-test and analysis of variance (ANOVA) when applicable. The relationship of lifestyle behaviours with the obesity indices were determined by regression analysis. Because of co-linearity of some variables and given that (as described in the methods), some variables as insulin and apolipoproteins and the dietary intake of energy and fat were available from the subsamples of the participants, we used univariate linear regression analysis for each variable. Logistic regression analyses were applied for the determination of the odds ratio of socioeconomic factors and lifestyle behaviours on obesity (BMI >30). Linear and logistic regression analyses were conducted separately in each gender after adjustment for age. The correlation of cardiometabolic risk factors with different obesity indices was calculated by bivariate correlation.

All statistical analyses were performed using the SPSS for Windows (version 13.0) (SPSS Inc., Chicago, IL). The p value of <0.05 was considered significant.

### Ethics

Ethics committees and other relevant national regulatory organizations approved the study. Written informed consent was obtained from the participants after full explanation of the study protocol.

## RESULTS

The study included 6,123 men and 6,391 women; their mean age was 38.89±14.93 years. The mean BMI was 26.62±5.51 for women and 24.55±4.62 for men (p<0.01). The mean WC and the mean WHR were 88.39±11.93 cm and 0.90±0.09 in men and 92.62±14.18 cm and 0.91±0.10 in women. These values were not significantly different in terms of gender.

Table 1 presents the anthropometric indices according to sex and age-groups. BMI, WC, and WHR increased with age until the sixth decade; thereafter, they declined in both males and females. The relationship of the obesity indices and lifestyle behaviours according to gender are presented in Table 2. It shows that current and passive smoking had an inverse significant association with BMI among males whereas current smoking, transportation by a private car, and longer duration of watching TV had a weak positive association with BMI, and GDI had an inverse weak association with BMI among females. Similar analysis for the assessment of the relationship of lifestyle behaviours with WC revealed that current and passive smoking, cycling, and GDI had an inverse weak association with WC among males. Higher consumption of fruits and vegetables, current and passive smoking, duration of daily sleep, and GDI had an inverse weak association with WC among females, and ex-smoking had a positive weak association with WC. Using a private car for transportation had a significant positive association with WHR among both the genders. Moreover, passive smoking had an inverse association with WHR among males, and the longer duration of watching TV had a positive association with WHR among females.

**Table 1.** Mean±SD of obesity indices based on gender and age-groups: IHHP

Age-group (years)	Total mean±SD			Males mean±SD			Females mean±SD		
	BMI	WC	WHR	BMI	WC	WHR	BMI	WC	WHR
19–29*	23.5±4.7	84.2±12.0	0.8±0.0	22.9±4.4	82.5±10.4	0.8±0.0	24.2±4.9	85.9±13.3	0.8±0.0
30–39†	26.0±4.8¶	90.8±12.4	0.9±0.0	24.8±4.1**, ¶	88.3±10.5	0.8±0.0	27.0±5.1¶	93.0±13.5	0.9±0.1
40–49‡	27.1±5.4§, **	94.5±12.5¶	0.9±0.0	25.8±5.2§, **, ¶	92.1±11.5**, ¶	0.9±0.0	28.3±5.4§, **	96.6±13.0§, **, ¶	0.9±0.0
50–59§	27.2±4.6‡, **	96.4±12.7**, ¶	0.9±0.0**	26.2±4.0‡, **, ¶	94.7±11.9**, ¶	0.9±0.0**, ¶	28.3±4.9‡, **	98.2±13.3‡, **, ¶	0.9±0.0**
60–65**	26.9±6.2‡, §	96.1±12.2§, ¶	0.9±0.0§, ¶	25.5±4.6†, §, ¶	93.3±10.8‡, §, ¶	0.9±0.0§, ¶	28.2±7.0‡, §	98.5±12.9‡, §, ¶	0.9±0.0§, ¶
≥65¶	25.6±5.2†	94.8±13.3‡, §, **	0.9±0.0**	24.7±4.2†, **	92.9±12.5‡, §, ‖	0.9±0.0§, **	26.7±4.6†	97.2±13.8‡, §, **	0.9±0.1**
	p<0.01	p<0.01	p<0.01	p<0.01	p<0.01	p<0.01	p<0.01	p<0.01	p<0.01

*,†,‡,§,**,¶Means with a common superscript symbol/sign have no significant difference;

BMI=Body mass index;

IHHP=Isfahan Healthy Heart Programme;

SD=Standard deviation;

WC=Waist circumference;

WHR=Waist-to-hip ratio

**Table 2.** Relationship of obesity indices with lifestyle behaviours according to gender: IHHP

Lifestyle factor	Body mass index				Waist circumference				Waist-to-hip ratio			
	Females		Males		Females		Males		Females		Males	
	ß	p value	ß	p value	ß	p value	ß	p value	ß	p value	ß	p value
Nutrition												
Global Dietary Index	−0.03	0.006	−0.02	0.05	−0.03	0.006	−0.03	0.01	−0.005	0.6	−0.01	0.3
Total kcal/day	0.02	0.3	0.02	0.4	0.03	0.2	−0.002	0.9	0.01	0.6	0.02	0.4
Total fat/day	−0.006	0.8	−0.03	0.3	−0.03	0.2	−0.07	0.3	0.002	0.9	0.02	0.4
Frequency of consumption of vegetables and fruits	0.01	0.3	−0.008	0.5	0.009	0.4	−0.037	0.006	−0.006	0.6	−0.01	0.2
Smoking status												
Current smoker	0.03	0.01	−0.05	<0.0001	0.02	0.02	−0.06	<0.0001	0.01	0.1	−0.02	0.07
Passive smoking	0.01	0.1	−0.3	0.01	0.02	0.04	−0.03	0.005	0.02	0.06	−0.041	0.001
Ex-smoker	0.005	0.6	−0.007	0.6	0.02	0.03	−0.01	0.4	0.01	0.3	−0.005	0.7
Physical activity												
Walking	−0.005	0.7	−0.009	0.5	−0.02	0.1	0.004	0.7	−0.07	0.6	−0.001	0.9
Cycling	−0.07	0.6	−0.003	0.8	−0.06	0.6	−0.04	0.2	−0.01	0.9	−0.02	0.2
Transportation	0.08	<0.0001	0.1	<0.0001	0.1	<0.0001	0.1	<0.0001	0.03	0.004	0.06	<0.0001
Leisure-time												
Daily sleeping	−0.03	0.003	−0.009	0.4	−0.4	<0.0001	−0.1	0.1	−0.02	0.06	−0.009	0.4
Watching television	0.04	<0.0001	0.004	0.7	0.01	0.3	0.01	0.3	0.03	0.4	0.002	0.8

ß: Standardized regression coefficient;

IHHP=Isfahan Healthy Heart Programme

Results of logistic regression analyses showed that living in urban area, being married, and having higher education level increased the OR of obesity among both the genders (Table 3). Non-manual work also increased this risk among males. Among lifestyle behaviours, the only significant variables were watching TV and current smoking that increased the OR for obesity among females.

**Table 3.** Logistic regression analysis of socioeconomic status and obesity* according to gender: IHHP

Socioeconomic parameter	Odds ratio	95% confidence interval
Males		
Urban vs rural residence	2.316	1.860–2.885
Secondary vs primary education	0.539	0.441–0.659
University vs primary education	0.427	0.304–0.600
Married vs single	3.493	2.530–4.824
Family members >4 vs <4	1.195	0.998–1.432
Income 25–75% vs <0.25%	1.134	0.906–1.418
Income >0.75% vs <0.25%	1.294	0.997–1.679
Ownership of car	2.044	1.711–2.442
Government job vs retired/not working	0.700	0.511–0.958
Private job vs retired/not working	0.599	0.482–0.744
Females		
Urban vs rural residence	2.596	2.290–2.943
Secondary vs primary education	0.502	0.442–0.570
University vs primary education	0.327	0.248–0.430
Married vs single	1.918	1.663–2.212
Family members >4 vs <4	1.184	1.046–1.340
Income 25–75% vs <0.25%	0.994	0.871–1.134
Income >0.75% vs <0.25%	1.050	0.889–1.241
Ownership of car	1.373	1.200–1.572
Government job vs retired/not working	1.253	0.917–1.712
Private job vs retired/housewife	0.916	0.711–1.181

*BMI >30;

BMI=Body mass index;

IHHP=Isfahan Healthy Heart Programme

We examined the correlation between the obesity indices and several metabolic risk factors (Table 4). Total and LDL-C, TG, FBG and apoB levels and total/HDL-C ratio, apoB/apoA ratio, and insulin resistance showed a significant correlation with all the three obesity indices. The association between the BMI and the apoB/apoA ratio was significant but such an association was not significant with WHR. A higher level of serum HDL-C had a significant correlation with lower levels of BMI and WC but not with WHR.

**Table 4.** Bivariate correlation of obesity indices and metabolic risk factors according to gender: IHHP

Biochemical variable	Body mass index		Waist circumference		Waist-to-hip-ratio	
	Males	Females	Males	Females	Males	Females
Total cholesterol (mg/dL)	0.21**	0.27**	0.22**	0.29**	0.16**	0.23**
HDL-C (mg/dL)	−0.03*	−0.04**	−0.03*	−0.03*	−0.01	−0.01
LDL-C (mg/dL)	0.13**	0.21**	0.16**	0.23**	0.14**	0.19**
Total-cholesterol/HDL-C	0.22**	0.25**	0.23**	0.25**	0.16**	0.20**
Triglycerides (mg/dL)	0.32**	0.29**	0.32**	0.32**	0.21**	0.24**
Fasting blood glucose (mg/dL)	0.13**	0.12**	0.17**	0.13**	0.14**	0.10**
Apolipoprotein A (mg/dL)	−0.002	0.07*	0.01	0.03	0.04	0.03
Apolipoprotein B (mg/dL)	0.20**	0.29**	0.18**	0.20**	0.16**	0.14**
apoB/A	0.21**	0.23**	0.18**	0.17**	0.13**	0.11**
Insulin resistance	0.10*	0.15**	0.10*	0.10**	0.10*	0.07*

*p<0.05;

**p<0.01;

apoB/A=apolipoprotein B/A;

HDL-C=High-density lipoprotein-cholesterol;

IHHP=Isfahan Healthy Heart Programme;

LDL-C=Low density lipoprotein-cholesterol

## DISCUSSION

Our findings confirmed the association of key variables of behaviour, socioeconomic levels, and metabolic factors with obesity and revealed the gender differences in this regard. This provides a new vision about the relationship between the obesity indices and the socioeconomic and behavioural factors in the Eastern Mediterranean region, notably in the Middle East. This study has clearly shown that CVDs and their risk factors are related to unhealthful lifestyles or adverse physical or social factors. In our study, factors, such as GDI, smoking status, and duration of daily leisure-time and physical activity, were generally associated with the obesity indices. These indices were also positively associated with different criteria used for SES, e.g. living in urban area, education, ownership of car, family crowding, and income.

Among the three commonly-used obesity indices, BMI is the most routinely-recorded index because of the simple measurement of weight and height and the widespread use of these parameters in surveys. There is a considerable variation in the amount of abdominal fat mass in people with similar BMI which mandates the use of a measure which takes abdominal fat into account (14); thus, some recent studies have recommended using WC alone (15). Practically, what matters is the level that each of these indices can predict other risk factors for CVDs and also clinical outcomes of patients.

We found a significant association between all the obesity indices and the major risk factors for CVDs; of special concern was the association of total and LDL-C with WC. Similar studies yielded different results (6, 14–16). Results of several studies suggest that WC may be a better predictor of cardiovascular risk than BMI or WHR is (10, 17–22). The study of Nakamura *et al*. in Japan revealed that WHR might predict the overall risk factors for CVDs better than BMI and WC do (6). Some studies have shown that WHR has a stronger association with future myocardial infarction and other sequelae of atherosclerosis (14, 18–19), which may be related to a positive association of WC with risk factors for CVDs (19). We observed a stronger association between the BMI and the apoB/apoA ratio than for WHR and this ratio. Similar findings have been reported in another study (20); however, the strength of the association of different obesity indices with this ratio was inconsistent among different ethnic groups. The ethnic differences can explain, to some extent, the controversies between ours and other studies. Altogether, the apoB/apoA ratio has been shown to be in a linear association with cardiovascular risk factors in diverse groups (21), and our findings indicate that this association can be generalized to obesity indices, irrespective of the strength of the association.

The associations we found between the SES factors and the obesity indices were inconsistent, which suggest that most SES factors cannot be directly used as surrogate predictors of obesity indices (22).

The results of the present study showed significant differences in some factors associated with obesity among men and women; such differences should be taken into account for interventional programmes at the individual level and for providing long-term public-health policies. Understanding the differences between the factors associated with gain in excessive weight among men and women can help individuals of both the genders consider healthful lifestyle plans that give them the best opportunity of achieving and maintaining their ideal weight. Gender is suggested to be a consistent moderator of relationships between BMI and psychological functioning (23). This should be considered in the modification of recommendations for lifestyles for achieving more healthful weight status. For instance, we found that, in our community, the duration of watching TV significantly increased the risk of obesity among females. So, instead of focusing on increasing physical activities and leisure-time in special classes outside the home, women can be encouraged to reduce the time spent on watching TV. In many communities, opportunities for different physical activities and sports recreation are available for males; as a result, females may face barriers that limit their access to, and participation in, outdoor physical activity and sports. Consequently, recommendation on reducing sedentary activities would be a more practical and successful strategy for the prevention and control of excessive weight in females than recommendation to attend outdoor physical activities.

We suggest that all the three obesity indices (BMI, WC, or WHR) should be considered to assess the risk of cardiovascular conditions and for follow-up purposes in epidemiologic studies.

### Limitations

The main limitation of this study is its cross-sectional nature; therefore, the associations documented in the statistical analysis should be interpreted with caution and should be confirmed in future longitudinal studies with long-term follow-up. The other limitation is the recall bias for the process of recalling and recording food intake and habits of physical activity. Moreover, considering the large number of individuals studied, the three-day food recall for estimating the precise nutrient and energy intake of the participants and some new factors for CVDs could be determined only in a subsample of the participants.

### Conclusions

The association of different socioeconomic and lifestyle factors and their gender differences should be considered for culturally-appropriate intervention strategies to be implemented at the population level for tackling obesity and associated cardio-metabolic risk factors. It should also be considered in individual counselling of healthcare providers who consult patients about how obesity and different related socioeconomic and lifestyle factors are connected to the risk of chronic diseases, notably CVDs and diabetes. Environmental and personal factors have an important influence on lifestyle behaviours and, in turn, on the weight status. Therefore, the findings of this study might be generalized to populations with similar socioeconomic and cultural backgrounds and can also be materialized through culturally-appropriate national and regional programmes and policies.

## ACKNOWLEDGEMENTS

The study is part of the Isfahan Healthy Heart Programme supported by Grant No. 31309304 from the Iranian Budget and Programming Organization in the Department of Health of the Ministry of Health and Medical Education, Isfahan Cardiovascular Research Center, and Isfahan Provincial Health Centre, both affiliated to the Isfahan University of Medical Sciences.
